# Facile Microwave-Assisted Hydrothermal Synthesis of Copper Oxide Nanoneedle Arrays for Practical Biomedical Applications

**DOI:** 10.7759/cureus.51678

**Published:** 2024-01-04

**Authors:** Veerappan Pradeep, Pitchaimani Veerakumar, Vishnu Priya Veeraraghavan

**Affiliations:** 1 Centre of Molecular Medicine and Diagnostics, Department of Biochemistry, Saveetha Dental College and Hospitals, Saveetha Institute of Medical and Technical Sciences, Saveetha University, Chennai, IND

**Keywords:** medicine, anti-oxidant, public health care, anti-inflammatory, nanotechnology

## Abstract

Introduction: Copper oxide nanoneedle arrays (CuO NAs) have been widely used as antibacterial agents and in therapeutic applications because of their unique physicochemical features, low cytotoxicity, low cost, exceptional antibacterial action, and significant interest in biomedicine. Various analytical techniques were used to assess the related phase constitution, optical characteristics, elemental content, and surface morphology. The X-ray diffraction (XRD) patterns and field-emission scanning electron microscopy (FE-SEM) micrographs revealed that the CuO NAs had a monoclinic phase with a nanoneedle-like shape. Our findings may cover the progress of innovative and effective anti-bacterial capabilities based on CuO NAs, which have been shown to be effective against various pathogens, making them ideal options for fighting bacterial infections.

Objective: This research aimed to synthesize CuO NAs using microwave-solvothermal (MW-ST) technology, explore their effectiveness, and assess their biological activity.

Methods: The CuO NAs were synthesized using the MW-ST process, and their properties were assessed using X-ray diffraction (XRD), Fourier transform infrared spectroscopy (FT-IR), field emission scanning electron microscopy (FE-SEM), energy dispersive analysis (EDS), field emission transmission microscopy (FE-TEM), and ultraviolet-visible (UV-Vis) techniques. The biocompatibility of CuO NAs was determined through hemolytic assays, and their bioactivities like antioxidant and anti-inflammatory assays were also determined.

Results: The CuO NAs were successfully developed, and various analytical tools were used to characterize and validate their morphology, size, crystallinity, and elemental compositions. It has been shown in in-vitro investigations that a strong anti-inflammatory impact is demonstrated by the inhibition of protein denaturation with low hemolytic potential. As a result, CuO NAs have the potential to be an excellent choice for anti-inflammatory solicitations.

Conclusion: CuO NAs were synthesized and characterized with various advanced techniques, revealing the formation of nanoneedles-like morphology. Based on the experimental findings, CuO NAs have the potential for anti-microbial, anti-oxidant, anti-inflammatory, and anti-hemolytic activities. However, additional in-vivo testing is essential to properly evaluate their efficiency and safety.

## Introduction

In recent years, copper oxide nanoneedles arrays (CuO NAs) have fascinated substantial interest in a wide range of applications in sensors, energy storage, photocatalysis, pharmaceuticals, therapeutic systems, and biological applications [1]. This is due to their inherited characteristics, which include a wide surface area, excellent biocompatibility, low toxicity, customizable size and shape, water-solubility, chemical inertness, and the ability to minimize toxicity by managing drug release to the human body [2]. CuO NA nanostructures have also received a lot of interest. It is a significant low-cost semiconductor material with a small band gap (~1.2 eV) that has prospective uses in a variety of industries [3].

Using copper (Cu) and its oxide-based nanomaterials is of great interest because of their active low-cost component, which displays strong redox behavior (easily oxidizes to convert CuO or Cu_2_O); specifically, it plays a vital role in the human being's metabolism process [4]. Various methodologies have been developed to synthesize CuO with various morphologies, such as nanoparticles (NPs), nanotubes (NTs), nanowires (NWs), nanorods (NRs), nanoplates (NPLs), nanolayers (NLs), nanoflowers (NFs), nanospheres (NSs), and nanourchins (NUs), being investigated for novel applications. The microwave-solvothermal (MW-ST) synthesis approach not only makes rapid heating of the reaction medium possible but also significantly reduces the reaction time during the whole procedure. Notably, MW synthesis is a great technique for producing CuO NPs, alloys, and core shells owing to its advantages, including a fast reaction rate, exceptional thermal efficiency, and a rapid energy supply. Furthermore, we found that the synthesis of CuO NAs may be enhanced to produce distinct morphologies by combining MW heating with the ST approach [5].

Antibiotics accumulate in the body through absorption in the gastrointestinal tract after oral administration, reaching peak concentrations in the bloodstream. They may also be distributed to various tissues, where they exert their therapeutic effects. Elimination occurs primarily through renal excretion, ensuring gradual clearance from the body. They can also accumulate in food when administered to animals in agriculture for disease prevention or growth promotion, leading to residual traces in meat and milk that enter the human food chain. Antibiotics have been frequently used in veterinary medicine and the human healthcare system because of their potent anti-bacterial properties and affordability. However, improper use or an excess amount may result in their accumulation in the body or in food, which has detrimental consequences for the public's health, including cytotoxicity, nephrotoxicity, allergic reactions, and bacterial resistance [6]. Therefore, developing an easy, sensitive, rapid, and selective analytical method for monitoring the antibiotic levels in food and the human body is crucial.

In recent years, metallic nanoparticles have frequently been employed as antibacterial and antifungal agents, yet there are still unresolved safety concerns for both humans and the environment in relation to their release and ingestion. For instance, excessive silver discharge harms humans and animals since it pollutes the ecosystem. Cu is no exception, as too much of it can cause the body to produce the most harmful radicals, including the hydroxyl radical [7]. Recently, Cu and CuO-based nanomaterials possess unique anti-bacterial and anti-fungal properties, which can initiate anti-microbial activity even without the presence of light [8]. Notably, Cu-transporting adenosine triphosphatases (Cu-ATPases) are of particular importance because they allow excess copper to be exported through the intestine (ATP7A), liver (ATP7B), and mammary gland (ATP7B), resulting in the production of milk [9].

Cu is more accessible than more expensive noble metals such as silver and gold, has a suitable redox potential, and high solution stability, and can be targeted in both in-vivo and in-vitro conditions, rendering it a better biomaterial for bioactivities. Apart from the aforementioned characteristics, CuO-based nanomaterials have been reported to have an antibacterial action against a number of species of bacteria and fungi [10]. A previous study has shown that the antibacterial property of CuO-NPs is produced through an electrochemical reduction method using surfactant as a structure-directing agent in an organic medium. The CuO-NPs were tested for antibacterial activity against human pathogens like *Escherichia coli* (*E. coli*) and *Staphylococcus aureus* (*S. aureus*) strains with satisfactory results. However, this method requires severe reaction conditions such as toxic organic solvents, complicated procedures, and difficulties in large-scale synthesis [11].

This research aimed to synthesize CuO NAs by using a simple MW-ST approach. Structural properties and chemical composition were analyzed with X-ray diffraction (XRD), scanning electron microscopy-energy dispersive analysis (SEM-EDS), field emission transmission microscopy (FE-TEM), Fourier transform infrared spectroscopy (FT-IR), and ultraviolet-visible (UV-Vis) spectroscopy. The biological activities of the as-prepared nanomaterials were studied, including antimicrobial, anti-inflammatory, antioxidant, and hemolytic assay actions.

## Materials and methods

Chemicals and reagents

Copper(II) nitrate (Cu(NO_3_)_2_. xH_2_O, 99.999% trace metals basis), polyvinylpyrrolidone (PVP, average molecular weight 40,000), ethylene glycol (EG), 2,2-diphenyl-1-picrylhydrazyl (DPPH), sodium hydroxide (NaOH pellets), and phosphate-buffered saline (PBS, 0.05% TWEEN® 20, pH 7.4) were procured from the Sigma-Aldrich chemical company (Burlington, Massachusetts, United States). All other chemicals were used with analytical purity in the experiment, and Milli-Q water was used throughout the work.

Preparation of CuO NAs

**Figure 1:**
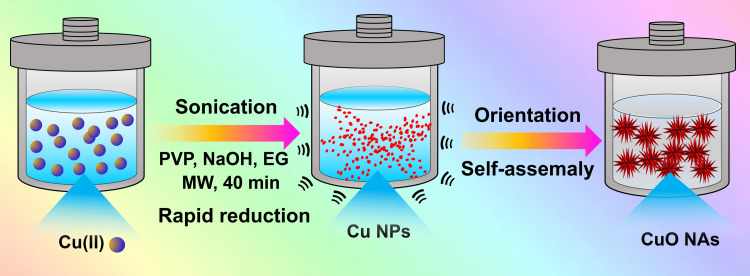
The preparation of copper oxide nanoarrays by the microwave-solvothermal process. PVP: Polyvinylpyrrolidone; MW: Microwave; EG: Ethylene glycol; NaOH: Sodium hydroxide

In a typical synthesis, Cu(II) salt (~100 mg), PVP (~50 mg), and NaOH (~25 mg) were dissolved in 40 mL of EG, and the whole mixture was sonicated for at least 30 minutes. The aforementioned precursor was placed in a 50 mL Teflon-lined stainless steel autoclave tightly sealed and it was subsequently heated at 180°C for 40 minutes. The autoclave could naturally cool to ambient temperature when the reaction was completed. The final products were collected and rinsed three times with Milli-Q water. The overall preparation procedure for CuO NAs is illustrated in Figure 1.

Characterization

The XRD pattern of CuO NAs was examined using an X-ray diffractometer of typical Cu-K radiation at a scan rate of 0.05°/min in the 20-90° range. An FT-IR spectrum was recorded in attenuated total reflectance (ATR) mode with a Bruker IR spectrometer (Billerica, Massachusetts, United States) in the region 4000-500 cm^-1^. The surface morphology was examined using scanning electron microscopy (JEOL JSM-IT800 SEM, Tokyo, Japan) and energy EDS with the JSM-IT800 SEM apparatus equipped with a silicon drift detector (Oxford X-MaxN 50 mm^2^, Oxford Instruments, Abingdon, Oxfordshire, United Kingdom). UV-vis spectroscopy (Thermo Scientific Evolution 220 spectrophotometer, Thermo Fisher Scientific, Waltham, Massachusetts, United States) was employed to measure the absorption study.

Antioxidant activity

It is a rapid and popular test for determining the anti-radical activity of nanomaterials, expressed as IC_50_, which denotes the concentration of each sample required to scavenge 50% of DPPH free radicals using ascorbic acid as a reference. DPPH is a typical nitrogen-concentrated free radical that is frequently employed to lessen the compound or vegetation extraction process's intense scavenger function. When hydrogen or electrons are accepted, the standard DPPH intensity is reduced. The DPPH assay is based on measuring the antioxidant capacity of CuO NAs to scavenge the DPPH radical. The reduced activity of the samples was determined by altering the color of the DPPH solution from blue/purple to yellow [12]. Typically, 2.0 mL of an alcoholic solution containing 0.1 mM DPPH was combined with 1.0 mL of different CuO NA concentrations. The obtained whole mixture was incubated in the dark condition at least for 20 min at room temperature. The absorbance is measured at 517 nm against a control consisting of 1.0 mL 2.0 mL methanolic solution of DPPH. The percentage of DPPH radical scavenging activity was calculated using the following formula: % Antioxidant activity = ( A0-A1)/A0 100%, where A0 is the absorbance of the control, and A1 is the absorbance of the extractives/standard.

Antimicrobial activity

The as-prepared CuO NAs were evaluated for their ability to inhibit the growth of the pathogens *E. coli, Klebsiella pneumonia *(*K. pneumonia*)*, S. aureus*, and *Candida albicans *(*C. albicans*) using the Agar well diffusion method. Here, dimethyl sulfoxide (DMSO) was used to suspend nanomaterials at a concentration of 100 mg/mL. Then, 25 and 100 mL of CuO NAs were added to each well and the mixture was incubated for 18-24 hours at 37 °C. The standard antibiotic discs of ampicillin, chloramphenicol, gentamicin, and fluconazole were used as positive controls for E. coli, K. pneumonia, S. aureus, and C. albicans, respectively. After an overnight of incubation, the zone of inhibition in diameter was measured. 

Hemolytic activity

Hemolysis refers to the release of hemoglobin into the plasma because of the erythrocyte membrane being damaged. Rehman et al.'s [13] approach was slightly modified in order to conduct the hemolysis activity test against nanomaterials. Typically, 1.0 mL of 3.8% sodium citrate was added to 9.0 mL of the blood sample to prevent blood clotting. It was then centrifuged in a centrifuge tube for five minutes at 3000 rpm. The pellet containing RBCs was re-suspended in 10 mL of PBS at a pH of 7.4 and the supernatant containing platelet-poor plasma was discarded.

The prepared CuO NAs were loaded with different concentrations (12.5, 25, 50, 100, and 200 g/mL) in five test tubes, then, each test tube was gently inverted after receiving 2 mL of erythrocyte suspension. After that, the tubes were gently shaken to maintain blood contact with the nanoparticle and incubated for 90 minutes at 37 °C. The same volume of erythrocyte solution was added to Triton X-100 and PBS (pH 7.4), respectively, to create positive and negative controls. After incubation, the samples were centrifuged for five minutes at 3000 rpm to remove the RBC cells. The supernatant was then carefully separated out and used for absorption studies at 540 nm using a UV-vis spectrophotometer against a PBS blank solution. The percentage of hemolytic index (%) was calculated by using the following formula: (%) Hemolysis =((absorbance of test sample)-(absorbance of diluent))/((absorbance of positive control)-(absorbance of diluent)) 100.

Anti-inflammatory assay

The in-vitro anti-inflammatory efficacy (inhibition of protein denaturation) was evaluated based on previous reports [14,15]. Typically, 50 μL of as-prepared CuO NAs solution was diluted with 450 μL of 5% weight/volume bovine serum albumin (BSA) protein before being incubated at 37 °C for 20 minutes and then heated at 60 °C for 3 minutes. The percentage of inhibition was calculated with the following formula: BSA Denaturation inhibition (%) = (A control - A sample)/A control ×100, where A control is the absorbance of the control and A sample is the absorbance of the test sample.

Statistical analysis

Each experiment was carried out three times and was set up using a completely random block design. The results are shown as the mean and SD. GraphPad Prism 5 (version 6, Dotmatics, Boston, Massachusetts, United States) was used to do the statistical analysis. The one-way analysis of variance (ANOVA) Dunnett's multiple comparison test's p-value (p < 0.05) was used to determine the significance of the experiment.

## Results

**Figure 2:**
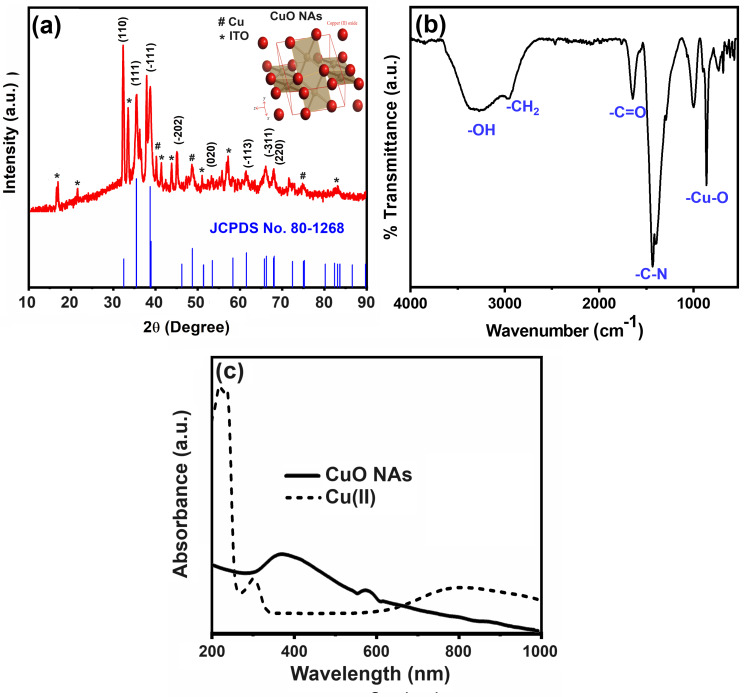
(a) X-ray diffraction pattern (# and * represents the metallic Cu and indium-tin oxide (ITO) peaks, respectively), (b) Fourier transform infrared spectrum, and (c) Ultraviolet–visible absorption spectrum of the copper oxide nanoneedles. JCPDS: Joint Committee on Powder Diffraction Standards

The XRD pattern of the as-prepared CuO NAs sample is presented in Figure 2a. Obviously, all the diffraction peaks are clearly well matched with the monoclinic CuO phase, according to the standard data (Joint Committee on Powder Diffraction Standards (JCPDS): 05-0661) [16]. The XRD pattern clearly shows the mixture of CuO and metallic Cu phases. The symbol * represents peaks of indium-tin oxide (ITO). Figure 2b shows the FT-IR spectrum of the synthesized CuO NAs with strong absorption bands ranging from ~3250 to 3480 cm^-1^, which are ascribed to the chemisorbed water molecules on their surfaces. The peaks observed at ~1443 cm^-1^ and ~1012 cm^-1^ correspondingly show different organic functional moieties wrapping over CuO NAs. The vibrational characteristics of stretching the Cu-O bond in monoclinic CuO NAs are shown by the absorption bands detected between 700-400 cm^-1^ [17]. Furthermore, the UV-vis absorption spectra of the Cu(II) and CuO NAs are displayed in Figure 2c. It can be observed that the broad absorption shows the electronic transition of Cu(II) with a maximum of 810 nm and several bands with dotted lines. In comparison, the solid line is the characteristic surface plasmon resonance (SPR) absorbance at the wavelength of 375 nm, which was confirmed by the formation of CuO NAs [18].

**Figure 3:**
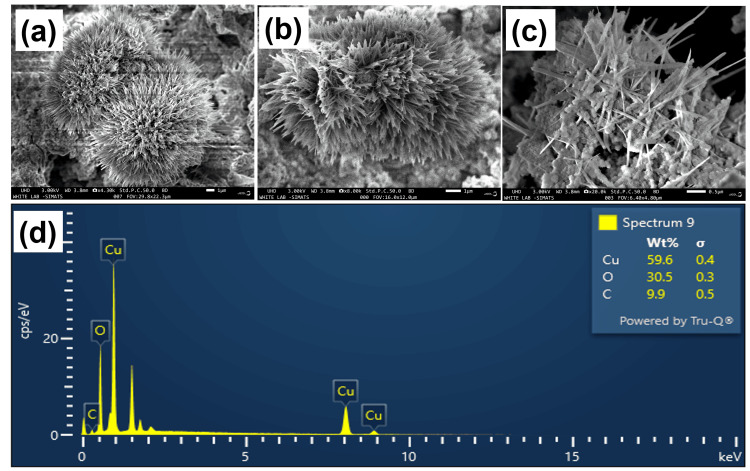
(a-c) Field emission scanning electron microscope images and (d) Energy dispersive X-ray spectroscopy spectrum of the copper oxide nanoneedles.

The morphology of the CuO NAs was analyzed by FE-SEM. Figures 3a-3c show typical FE-SEM images of as-prepared CuO NAs nanostructure exhibiting a needle-like structure with a high density, indicating that each nanoneedle consists of a wide stem and a sharp tip. The precise structure and dimensions of CuO NAs were assessed using a high magnification FE-SEM picture (Figure 3c), with a half-length width ranging from 0.5 to 1 μm, demonstrating a more uniform size and shape. The EDS spectra show peaks relevant to Cu and oxygen (O) signals, confirming the formation of CuO NAs (Figure 3d). The carbon signal emitted by the carbon tape is notable. The quantities of Cu and O elements are evaluated from the corresponding EDS analysis (Figure 3d inset), confirming that the CuO NAs are composed of only Cu and O atoms.

**Figure 4:**
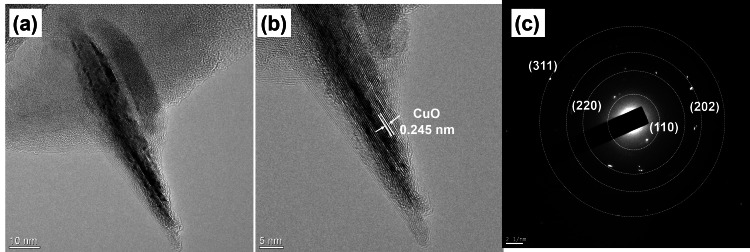
(a,b) Field emission transmission electron microscope images and (c) Selected area electron diffraction pattern of copper oxide nanoneedles.

Examination of CuO NAs by FE-TEM revealed that they appeared as nanoneedles, made of sharp tips with smooth surfaces, which is in line with our FE-TEM observations and indicated that the lattice fringes distance is about 0.245 nm (Figure 4a and Figure 4b). The selected area electron diffraction (SAED) analysis collectively revealed the crystalline nature of CuO NAs are in a monoclinic phase (Figure 4c).

Antimicrobial activity

**Figure 5:**
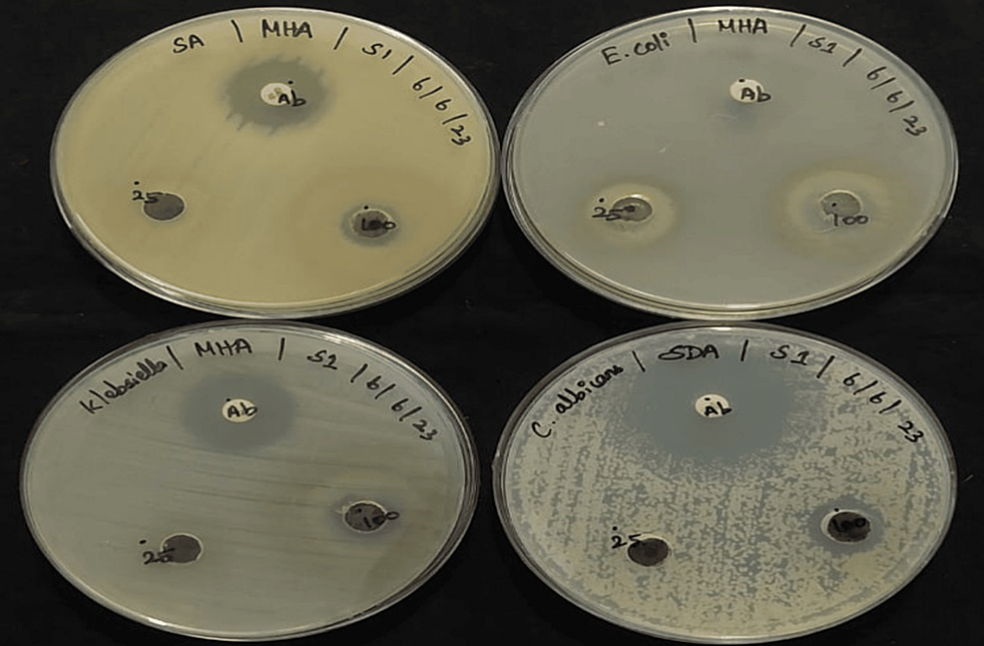
Antimicrobial activity of copper oxide nanoneedles against various pathogens: Escherichia coli (E. coli), Klebsiella pneumonia (K. pneumonia), Staphylococcus aureus (S. aureus), and Candida albicans (C. albicans).

The CuO NAs antimicrobial action was observed against the predominant opportunistic human pathogens such as *E. coli*, *K. pneumonia*, *S. aureus*, and *C. albicans; *the corresponding ampicillin, chloramphenicol, gentamicin, and fluconazole were used as reference drugs and to compare the efficacy of CuO NAs' antibacterial properties. We tested anti-bacterial activity in a dose-dependent manner using the CuO NAs, as displayed in Figure 5. The antibacterial activity was very low (<1 mm) against *E. coli, K. pneumonia, and S. aureus* (i.e., minimum zone of inhibition was observed at a concentration of 25 µg/mL. In the case of 25 µg/mL, around 13 mm zone of inhibition (ZOI) was observed for all the tested bacterial pathogens such as *E. coli, K. pneumonia, *and *S. aureus*; whereas, 17 mm ZOI was noted for *C. albicans*. Purines or ionic channels may also be used to enter the cell. However, it is unclear exactly how they transfer across the cellular wall.

As reported in the literature, *C. albicans* belongs to the fungi group whose cell wall is made up of chitin, which is the strongest of the more permeable Cu^2+^ ions. The above results reveal that CuO NAs are proven to have strong anti-fungal action against *C. albicans* in clinical isolates, which indicates that the CuO NAs exhibit greater activity against all microbes at 100 µg/mL, while compared to the *E. coli and Klebsiella sp.* (Figure 5). At the application of a higher concentration of CuO NAs (100 µg/mL), a greater bactericidal effect of CuO NAs was observed compared to 25 µg/mL of concentration. The reason for growth inhibition can be the possible interaction between the external membrane of bacteria and the CuO NAs. CuO NAs may disrupt the integrity of cell membranes causing malfunctioning of enzymes and increasing cell permeability leading to bacterial cell death [19]. Also, it is evident that CuO NAs exhibit stronger anti-bacterial activity against the studied bacterial strains because of their excellent stability in the growing medium, which promotes more interactions between bacterium and CuO NAs.

Antioxidant activity

**Figure 6:**
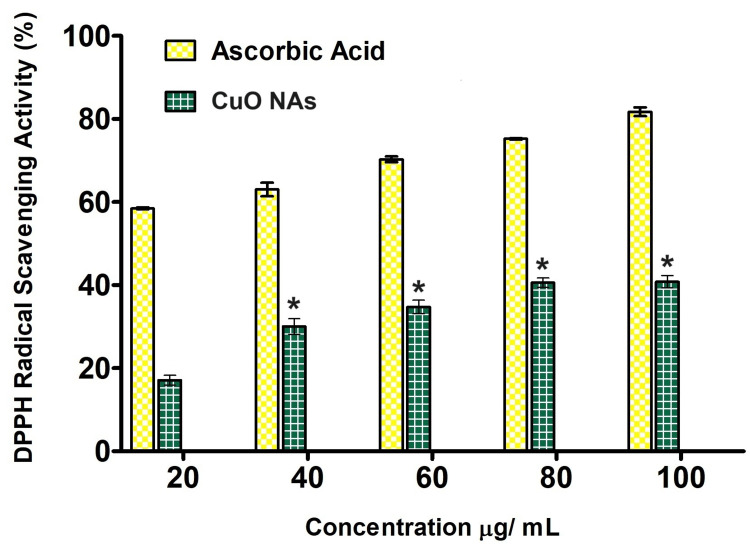
The antioxidant property of copper oxide nanoneedles using 2,2-diphenyl-1-picrylhydrazyl (DPPH) radical scavenging potential with different concentrations (n = 3). The experiment was performed in triplicates and values are expressed in mean ± SD. *Values are statistically significant from the group incubated (P < 0.05).

The DPPH scavenging activity of CuO NAs is shown in Figure 6. The potential of ascorbic acid (AA) to scavenge DPPH radicals is directly proportional to the concentrations. DPPH radical scavenging activity of CuO NAs and standard AA is presented in Figure 6. The cumulative effect of CuO NAs shows maximum efficiency, which is 18.48 ± 0.18%, 30.12 ± 0.15%, 38.62 ± 0.25%, 41.15 ± 0.18%, and 41.28 ± 0.12% in DPPH radical scavenging activity at 100 µg/mL, whereas the standard AA exhibit 80.48 ± 0.45% activity. The lower values reflect the greater potency for antioxidant activity of the CuO NAs in comparison to bulk CuO [20]. The inhibition of DPPH activity assumes the existence of a linear relationship (with should also be validated) between DPPH activity and CuO NAs concentration (Figure 6) the relationship could be magnified by coefficient correlation and also be tested by p-value and it was compared with AA.

Anti-inflammatory assay

**Figure 7:**
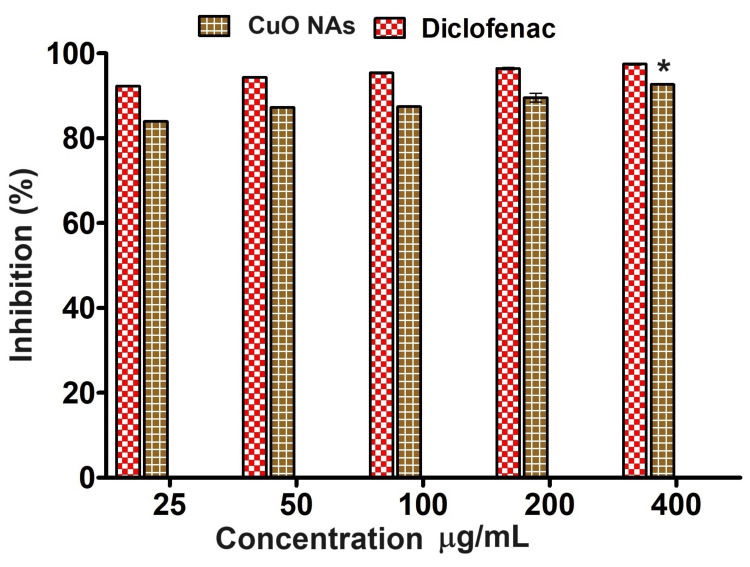
Anti-inflammatory activity of copper oxide nanoneedles. The experiment was performed in triplicates and values are expressed in mean ± SD. *Values are statistically significant between different concentrations (P < 0.05).

Inflammation is caused by the abnormal production of cellular lysosomal enzymes, which leads to the development of cancer [21]. The results are compared with standard diclofenac as a positive control. According to the results of an in vitro anti-inflammatory experiment, CuO NAs significantly and dose-dependently decreased the denaturation of BSA (Figure 7). The experiment was performed in triplicates and values are expressed in mean ± SD. This shows that the CuO NAs may reduce inflammation and help develop new anti-inflammatory drugs.

Hemolytic assay

**Figure 8:**
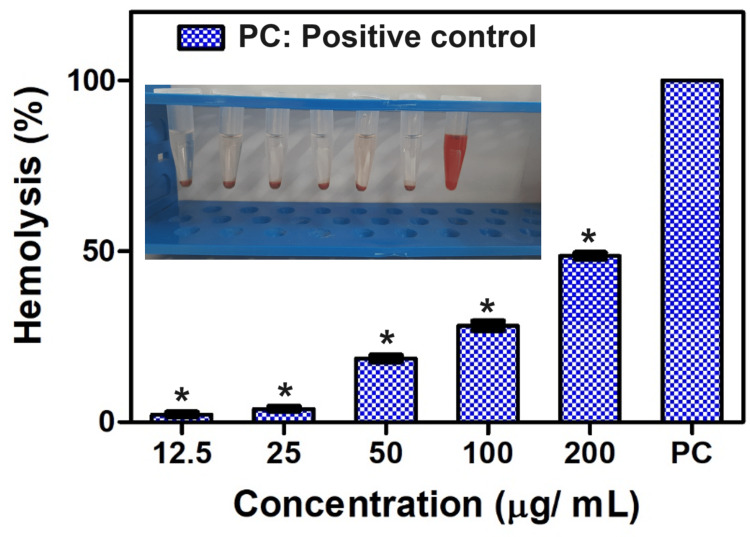
Hemolytic activity of copper oxide nanoneedles. The experiment was performed in triplicates and it was expressed as mean ± standard deviation (SD). *Values are statistically significant compared to the control group (P<0.05).

In comparison to the control, the CuO NAs demonstrated less than 5% hemolysis in erythrocytes at a lower concentration of 12.5 μg/mL. The hemolytic assay test results are shown in Figure 8. However, hemolysis increased as a function of increasing concentrations of CuO NAs from 25, 50, and 100, to 200 μg/mL. The results illustrated the dose-dependent raising protective action of CuO NAs with a gradual increase in the concentration from 25 to 200 µg/mL. These encouraging results of CuO NAs with significant protective action intern low hemolysis percentage show the manifestation of hemocompatibility of CuO NAs towards human red blood cells [22].

## Discussion

In this present work, the XRD patterns were detected to be at 2θ = 32.38, 35.65, 38.71, 48.75, 53.54, 58.35, 61.59, and 66.34 assigned to (110), (111), (200), (−202), (020), (202), (−113), and (022) reflection lines, respectively, of monoclinic CuO NAs (JCPDS: 05-0661, Figure 2a). This study shows that the CuO NAs are monoclinic (crystalline) in nature and the average crystallite size is estimated by Scherrer's equation [23] from the full-width-half-maximum of the (110) peak of the XRD pattern. Based on the above Scherrer's equation, the average length of the needle shape is ~95 nm ± 5 nm and the diameter is 25 nm ± 5 nm. The FT-IR absorption peaks revealed the vibrational modes of CuO NAs in the range 700-400 cm^−1^ with stretching of Cu-O, which shows that the metal oxide peak suggests the formation of the CuO nanostructure (Figure 2b). The UV-visible spectrum of CuO NAs was recorded (Figure 2c), which gives a characteristic peak related to the SPR band of the CuO nanostructure. It was interesting to note that most of the CuO NAs in the SEM and TEM images were found to be nanoneedles arrays, as well as dispersed in nature. Typical diameters of the stem part and the sharp ultrathin nanotip range between 85 ± 15 and 20 ± 5 nm, respectively, and the whole length of the nanoneedle appears to be 200 nm to 0.5 μm with a more uniform distribution of arrays (Figure 3a-c). It is an array that produces hierarchical nanostructures because of its noticeably rough surface, large number of nanoneedles, and voids. The EDS spectrum for the CuO NAs confirmed Cu and O in the CuO NAs with no impurity peaks (Figure 3d). The FE-TEM analysis is shown in Figure 4, which describes the size and crystallinity of the synthesized CuO NAs, whereas the distinct bright concentric rings in the SAED pattern further confirm the crystallinity of CuO NAs.

The CuO NAs were exploited for hemolytic assay, and anti-microbial, anti-inflammatory, and antioxidant activities, owing to the high surface-to-volume ratio and remarkable biocompatibility [24]. The antibacterial activity of the synthesized CuO NAs against the Gram-negative bacterial pathogen (*E. coli*) was observed, with the highest ZOI, shown in Figure 5. The same type of highest-growth inhibitory result was reported in *E. coli* and *K. pneumonia* by Meghana et al. [25]. The CuO NAs' antifungal properties against a fungal strain (*C. albicans*) show the maximum sensitivity to CuO NAs, which is more powerful than that of the positive control (ampicillin) strain, defining a clear ZOI for conventional antifungals and destroying the growth of tested pathogens, which causes membrane damage to the fungi [26]. The CuO NPs have a large surface area, making them highly reactive. The distinctively high surface-to-volume ratio of CuO NPs permits them to interact with the cell membrane of the bacteria through their surface, resulting in the death of the bacteria [27]. Notably, with an increased DPPH radical impact, it is stated that MW-ST synthesized CuO NAs have more antioxidant properties, are non-toxic, and are environmentally friendly, as well as they are a prospective candidate for a variety of medicinal applications [28].

CuO NAs were applied in an additional investigation to evaluate protein denaturation compared to the non-steroidal anti-inflammatory drug diclofenac. CuO NAs have anti-inflammatory properties by promoting protein breakdown, according to the results of the protein denaturation assay. For CuO NAs, the range of protein denaturation inhibition is 81.5% to 91.3% at doses between 25 and 400 μg/mL (Figure 7). This outcome is comparable to the anti-inflammatory properties of diclofenac (94%) and metformin (85.9%) used to treat diabetes. Sultana et al. [29] have prepared an eco-friendly method for producing Cu NPs using plant leaf extract. The in-vitro anti-inflammatory properties of the synthesized Cu NPs showed the maximum inhibition of protein denaturation at 54.15% at a concentration of 400 µg/ml, whereas our CuO NAs exhibited 91.3% of protein denaturation at a dose of 400 μg/mL. Therefore, our findings provide a viable foundation for mitigating inflammation and protein breakdown in hyperglycemic situations. As shown in Figure 7, at all concentrations of CuO NAs, except at 25 µg/mL, hemolysis is below the 5% allowed by the International Organization for Standardization (ISO) 10993-4 [30]; therefore, concentrations below 25 µg/mL (Figure 7) can be considered hemocompatible. Based on these findings, it can be said that the use of CuO NAs has promising applications in biomedicine and other biological activities.

Recently, there have been efforts by researchers to modify the morphology or size of CuO to enhance biological activity. We could attempt a plethora of additional combinations. Additionally, CuO-based nanoparticles have become a viable option for obtaining enhanced and selective biological properties. The biological performance translates into more active sites, adaptability, and selectivity as a result of the synergistic effects. Therefore, more research in these fields is required to demonstrate the potential production and use of CuO-based biological activities in modern drug discovery. Finally, we think that further study is required to assess and possibly even relate the structure of the modifying material containing CuO NAs to the anticipated future performance of the developed biomedical studies.

Novelty of the work

So far, various silver (Ag)-, gold (Au)-, Cu-, and platinum (Pt)-based nanomaterials have been utilized for biomedical applications owing to their high electrical conductivity, high stability, large surface area-to-volume ratio, and biocompatibility. On the other hand, Cu offers many benefits over other nanomaterials, including low cost and being an essential trace metal in the human body that is mostly absorbed in the stomach and small intestine before being eliminated in the bile. Additionally, it plays a significant catalytic role and regulates many enzymes and proteins in the body through multiple mechanisms. The MW-ST approach is one of the most well-known and widely employed methods for the synthesis of diverse CuO nanostructured materials, including CuO NAs. This method is simple, cost-effective, eco-friendly, and easily controllable. These characteristics make it highly advantageous for the large-scale synthesis of CuO NAs. Herein, the CuO NAs were prepared by the MW-ST method and characterized with different sophisticated analytical tools and used for applications. The CuO NAs so prepared were utilized for anti-microbial, anti-oxidant, anti-inflammatory, and anti-hemolytic activities.

Limitation

This study explored the application of CuO NAs and addressed several biomedical functions with encouraging outcomes in laboratory-based practical studies. However, additional research is required to transition from controlled laboratory settings to practical, real-world scenarios. While most of our present research has been on immediate consequences, there is clearly a need to examine long-term impacts, considering the possibility of bioaccumulation. Another limitation is that a single antibiotic is chosen as a reference. Although hemolytic activity has been evaluated, the future research path has to include a more thorough cytocompatibility assessment that considers unintended consequences, the dosage of CuO NAs, and turning the form stability. Moreover, a thorough analysis of CuO Nas' interactions with bio-environments and adherence to regulatory requirements give a route for future research in medicine. Further studies need to be performed in natural conditions (in vitro or in vivo) to find the actual mechanism of CuO NAs in reducing inflammation. However, it is important to search for efficient CuO NAs, to use low concentrations for safety, and also to explore their cytotoxicity in different mammalian cell lines.

## Conclusions

In conclusion, we successfully synthesized the CuO NAs via a simple, affordable MW-ST approach. The XRD, FT-IR, FE-SEM, FE-TEM, EDS, and UV-Vis techniques were used for CuO NAs characterizations. The morphology of the nanoneedles was confirmed by FE-SEM and HR-TEM microscopy, while the XRD pattern revealed a monoclinic structure. We tested the antimicrobial activities using CuO NAs through the agar disk-diffusion method, and the results were more effective. Notably, the hemolytic assay revealed that CuO NAs are compatible with low doses and have the potential to be well-tolerated in therapies. In addition, the antioxidant and anti-inflammatory activity results showed that the CuO NAs perform better when compared to the standard. Moreover, CuO NAs can be exploited as a biocompatible nanomaterial in the fields of biomedicine, pharmaceuticals, nutrition, agriculture, and the environment because of their favorable antifungal activity.
